# Exercise interventions for the prevention of depression: a systematic review of meta-analyses

**DOI:** 10.1186/s12889-020-09323-y

**Published:** 2020-08-18

**Authors:** Mandy X. Hu, David Turner, Ellen Generaal, Daniel Bos, M. Kamran Ikram, M. Arfan Ikram, Pim Cuijpers, Brenda W. J. H. Penninx

**Affiliations:** 1 Department of Psychiatry, Amsterdam Public Health Research Institute, Amsterdam UMC, Vrije Universiteit Amsterdam, Oldenaller 1, 1081 HJ Amsterdam, The Netherlands; 2 Department of Clinical, Neuro and Developmental Psychology, Amsterdam Public Health Research Institute, Vrije Universiteit, Amsterdam, The Netherlands; 3 Department of Epidemiology, Erasmus MC University Medical Center Rotterdam, Rotterdam, The Netherlands; 4 Department of Radiology and Nuclear Medicine, Erasmus MC University Medical Center Rotterdam, Rotterdam, The Netherlands

**Keywords:** Depression, Physical activity, Systematic review, Meta-analysis, Randomized controlled trial

## Abstract

**Background:**

Exercise may be a promising target for depression interventions. However, evidence for a beneficial effect of exercise interventions on the prevention of depression differs substantially across different studies.

**Methods:**

A systematic search was performed up to July 2018 using PubMed, Embase, PsycINFO, and Cochrane. Articles were included if a meta-analysis of randomized controlled trials was performed that examined the effect of exercise interventions on the onset of depression or depressive symptoms in the general population. Meta-analyses focusing on treatment of diagnosed depression were excluded. Two authors independently screened the articles and graded the quality of included meta-analyses using AMSTAR 2.

**Results:**

Eight meta-analyses were included that showed little overlap in 134 included studies. All meta-analyses reported on depressive symptoms rather than onset of depression. Five of these were rated as moderate quality and three of low quality. Six meta-analyses found significant effects, and two found non-significant effects of exercise interventions in reducing depressive symptoms in children, adolescents, adults and the elderly (effect sizes ranging from − 0.10 to − 0.81). Scarce evidence did not allow to draw conclusions about the role of sex and characteristics of exercise on depression. However, some findings suggest that low intensity exercise was as effective as high intensity exercise. Heterogeneity among primary studies was high, likely caused by differences in study quality and exercise characteristics.

**Conclusions:**

The evidence from this study suggests that exercise interventions have a beneficial effect on depressive symptoms in the general population across a wide age-range.

## Background

Depression is one of the most common mental health disorders with a high burden of disease and the leading cause of years of life lost due to disability according to the World Health Organization [1]. This mental health disorder affects about 150 million people worldwide at any moment [2]. Approximately 1 in 5 women and 1 in 8 men will suffer from a major depressive episode during their life [3]. In addition to the large burden on quality of life, depressive disorder is also accompanied with a substantial economic burden [4]. Antidepressant use and psychotherapy have shown to be effective in the treatment of depression, although the effect sizes of treatments are generally modest and not all participants respond to these treatments [5]. Therefore, there is a growing need for strategies targeted at early interventions or ultimately even prevention of this psychiatric disorder [2]. One promising target for such interventions is exercise [6]. However, evidence for a beneficial effect of exercise interventions on the prevention of depression differs substantially across different studies [7, 8]. These differences may be explained by heterogeneity in study population and type of exercise intervention. For instance, the effect of exercise on depression may be modified by sex, age, or characteristics (e.g. mode and intensity) of exercise. Answering these questions of moderation is important, as it will inform the public about which type of exercise is effective for whom in the prevention of depression. Other moderators may also be relevant in this relationship, but the generic factors above are more likely to be measured and reported within meta-analyses compared to more specific factors. A comprehensive overview is needed of the evidence to date on the efficacy of exercise, and it is yet to be clarified whether these effects are modified by study and sample characteristics.

Cross-sectional studies have previously shown exercise and depression to be associated [9, 10], and prospective studies have indicated that low exercise may precede the onset of depression [11, 12]. In 2013, a systematic review on prospective observational studies was performed by Mammen and Faulkner [13] to investigate the effect of physical activity on the occurrence of depression. The authors concluded that exercise at any intensity level was likely to prevent subsequent depression. These results were corroborated by findings from a recent meta-analysis on prospective cohort studies by Schuch et al. [14], indicating that people with high levels of exercise had lower odds of developing depression. These articles, however, solely focused on prospective observational studies, which provide a good foundation to suspect causality, but are imperfect in proving it.

More convincing evidence that exercise may prove to be a valuable prevention strategy for depression comes from randomized controlled trials (RCTs) that investigated the effect of exercise interventions on the onset of depression and depressive symptoms in the general population [8, 15, 16]. Several meta-analyses have gathered these studies and assessed the overall efficacy of exercise interventions on depression, usually focusing on specific age-groups, such as children, adolescents, adults, or older adults [17–20]. In 2015, Rebar and colleagues [21] performed a meta-analysis of meta-analyses to investigate the effect of exercise on depression in general adult populations. They concluded from two meta-analyses that exercise reduced depression by a moderate effect. Although this work was comprehensive, the authors focused on adults and did not include studies focusing on children or adolescents, nor did they examine possible sex differences. In addition, the authors investigated the general effect of exercise on depression and did not elaborate on specific characteristics of exercise, such as mode, frequency, duration, and intensity. Furthermore, this study has been conducted some years ago and an update on the evidence in this field is timely.

This systematic review aims to give an overview of meta-analyses of randomized trials published from data inception to July 2018 on the effect of exercise interventions on depression and depressive symptoms. As the aim of this study was to investigate this from a preventive rather than a treatment perspective, meta-analyses were included that focused on general populations. Meta-analyses that only reported on patient populations or on specific populations with acute or chronic physical or mental illnesses were excluded. Additionally, the modifying effects of sex, age, and characteristics of exercise, such as mode and intensity were explored.

## Methods

### Literature search

The protocol for this systematic review can be found on PROSPERO (ref: CRD42018094215, available from https://www.crd.york.ac.uk/prospero/display_record.php?ID=CRD42018094215). This systematic review was conducted in accordance with the PRISMA guidelines. Two researchers performed the literature search on meta-analyses published up to July 2018 using the databases PubMed, Embase, PsycINFO, and The Cochrane Database of Systematic Reviews. Search filters were used that selected systematic reviews/meta-analyses. The search strings for each database can be found in Appendix A. The reference lists of relevant reviews were scanned to identify other articles of interest.

### Eligibility criteria

Two independent researchers (authors MXH and DT) screened the meta-analyses for inclusion, first based on titles and abstracts and then on full text. Disagreements were discussed and consensus was reached in all cases. Articles were included in the systematic review if they met the following criteria according to PICOS guidelines (participants, intervention, comparison, outcome, study design [22]): (1) Participants were general or at-risk populations at baseline. If both clinically depressed and general populations were included, the meta-analysis had to report separately on general populations in a sub-group analysis in order to be included; (2) Intervention: any type of exercise intervention; (3) Comparison: any type of comparison condition; (4) Outcome: onset of depression or depressive symptoms; (5) Study design: meta-analyses of RCTs published from data inception to July 2018. Meta-analyses were excluded if they: (1) only employed a systematic review or single study and no meta-analysis (no pooled effect across studies); (2) only reported on observational studies; (3) only reported on clinical populations with depression at baseline (determined by diagnosis or using depression threshold cut-off scores on self-report scales); (4) specifically focused on populations with acute or chronic physical or mental illnesses; (5) were published in a language other than English, Spanish, German, Dutch, or Greek (based on the language skills of the authors).

### Included meta-analyses

**Fig. 1.**
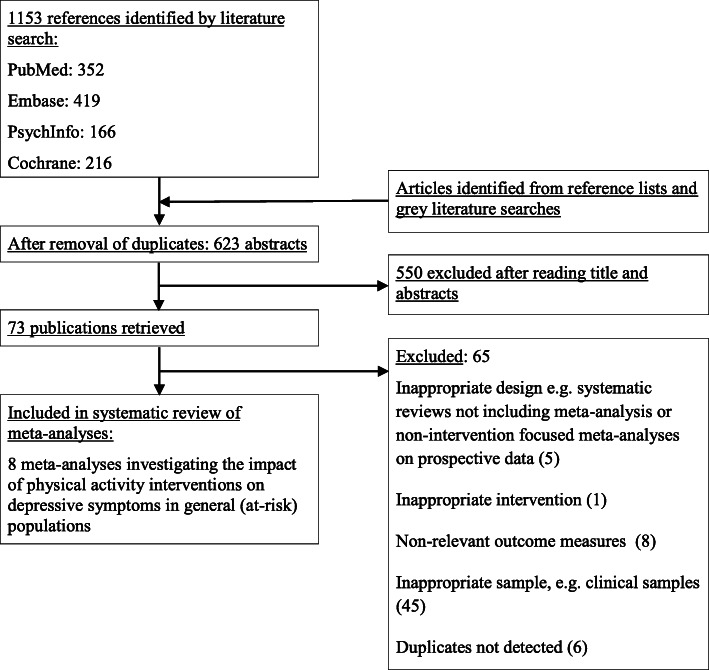
PRISMA flow diagram of the search and selection process for the meta-analyses included in the current study

A PRISMA flow diagram [23] of the search and selection process is presented in Fig. 1. The initial search resulted in 1153 publications. Removal of duplicates resulted in 623 remaining articles. After screening the publications by title and abstract, 550 articles did not meet the criteria, mainly because they did not employ a meta-analysis. The remaining 73 articles were read in full text and at this stage 65 articles were excluded, mostly because they focused on populations with clinical depression or physical or mental illnesses at baseline (*n* = 45). See Supplementary Table 1 for list of excluded articles and reasons. Finally, eight meta-analyses were included in this study.

### Quality assessment

Two raters (authors MXH and EG) independently graded the quality of the meta-analyses by using the 16-item AMSTAR 2 (A MeaSurement Tool to Assess systematic Reviews, revised instrument 2) [24]. Quality classifications were the following: critically low, low, moderate, and high quality. In case of classification divergence, the coders discussed the discrepancies in grading and reached consensus, which was not necessary (divergence: 0%).

### Data extraction

Data on relevant study characteristics and effect sizes (standardized mean difference (SMD)) from meta-analytic comparisons were extracted by one author (MXH). Information was extracted on the number of RCTs and the number of participants included in each comparison. Data on heterogeneity present in meta-analytic comparisons was also obtained. Extracted study characteristics were based on PICOS (participants, interventions, comparators, outcomes, and study design) criteria [22]. The descriptions given in Table 1 were extracted from the authors’ reports on the individual studies as well as from data reported within individual studies.

**Table 1:** Summary of meta-analyses included in the current study

Meta-analyses	Target population	Setting	Exercise intervention (mode, frequency, duration)	Control comparison	Depression outcome
Brown et al. 2013 [19]	Children and adolescents between 5–19 years	Most included studies targeted at-risk (clinically overweight young people or criminally institutionalized youth offenders) groups for depression.	Aerobics, fitness, yoga; 3–7 times/week; 12–28 weeks	Non-physical control group	Depressive symptoms measured by several depression rating scales
Carter et al. 2016 [25]	Adolescents between 13–17 years	Included studies targeted high school students from the general population.	Dance, yoga, other sports; 1–4 times/week; 9–40 weeks	Continued regular exercise or no exercise control group	Depressive symptoms measured by several depression rating scales
Conn 2010 [18]	Adults ≥18 years	Included studies targeted healthy adults without acute or chronic physical or mental illness. Studies targeting subjects with clinical depression or subjects that scored above a primary study specified depressive symptom criterion score were excluded.	Supervised and unsupervised exercise; mean of 3 times/week; 1 day – 1 year	Any control	Depressive symptoms measured by several depression rating scales
Forsman et al. 2011 [20]	Elderly people ≥65 years	Included studies targeted the general older adult population, older adults at risk for depression, or those who already suffer from subclinical symptoms of depression but did not fulfill the diagnostic criteria for a depressive disorder.	All types of exercise. No details provided.	Care as usual, waiting list, or no-intervention control	Depressive symptoms measured by several depression rating scales
Gordon et al. 2018 [26]	No criteria reported	Healthy population with no physical or mental illness	Resistance exercise training; 2–7 times/week; 6–52 weeks	No treatment, wait list, usual care, or patient education	Depressive symptoms measured by several depression rating scales
Larun et al. 2006 [27]	Children and adolescents ≤20 years	Included studies targeting general child/adolescent populations within all kinds of settings. Trials involving psychotic or borderline conditions, autism, physical handicap, eating disorders or chronic somatic diseases were excluded.	Running, aerobics, resistance training, fitness; 3 times/week; 6–40 weeks	Waiting list, non-intervention group, a low intensity exercise group or a psychosocial intervention group	Depressive symptoms measured by several depression rating scales
Park et al. 2014 [28]	Elderly people ≥65y	Included studies targeted elderly people who did not have disorders of orientation and who were capable of independent living.	Aerobics, walking, balance exercise, resistance training, Qigong, Tai chi; 1–3 times/week; 4 weeks – 1 year	No treatment/placebo or any other type of non-exercising intervention	Depressive symptoms measured by several depression rating scales
Rethorst et al. 2009 [17]	No criteria reported	Non-specified general population.	Aerobic or resistance training, or combined; 3–5 times/week; 4 weeks – 1 year	No-treatment or wait-list control	Depressive symptoms measured by several depression rating scales

*RCTs* randomized controlled trials, *Exercise* physical activity

## Results

### Characteristics of meta-analyses

A summary of the meta-analyses included in this systematic review can be found in Table 1. All eight meta-analyses reported on the effect of intervention promoting exercise on depressive symptoms. There were no meta-analyses that reported on the effect of exercise on the onset of depression, nor did any of the meta-analyses employ selection criteria that required samples to be entirely depression-free at baseline. Two meta-analyses included studies with children and adolescents (5–20 years) [19, 27], one included studies with adolescents (13–17 years) [25], two focused on elderly people (≥65 years) [20, 28], one focused on the adult population (≥18 years) [18], and two did not report an age-criterium [17, 26]. Although the meta-analyses included all types of exercise interventions, most interventions were based on aerobic or resistance training.

### Overlap of studies between meta-analyses

The meta-analyses included a total of 134 studies. Of these, 119 studies were cited once, 13 studies were included in two meta-analyses, and two studies were included in three meta-analyses. The most overlap (10 studies) was seen between the meta-analyses by Conn [18] and Rethorst et al. [17] (see Table 2 for percentage of unique studies per meta-analysis).

**Table 2:** Quality of meta-analyses according to AMSTAR 2 criteria

AMSTAR 2 criteria	Brown et al. 2013 [19]	Carter et al. 2016 [25]	Conn 2010 [18]	Forsman et al. 2011 [20]	Gordon et al. 2018 [26]	Larun et al. 2006 [27]	Park et al. 2014 [28]	Rethorst et al. 2009 [17]
Did the research question and inclusion criteria include components of PICO?	V	V	V	V	X	V	V	V
Were the review methods were established prior to the conduct of the review?	X	X	X	X	X	X	X	X
Did the authors explain their selection of the study designs for inclusion?	X	V	X	X	V	V	V	V
Was a comprehensive literature search strategy used?	V	V	V	V	V	V	V	V
Was study selection performed in duplicate?	X	V	X	V	X	V	V	X
Was data extraction performed in duplicate?	V	V	V	V	V	V	V	X
Was a list of excluded studies and justification for exclusions provided?	V	V	X	X	V	V	V	X
Were the included studies described in detail?	V	V	X	X	V	V	V	V
Was a satisfactory technique used to assess RoB?	V	V	X	V	V	V	V	X
Were sources of funding of the included studies reported?	X	X	X	X	X	X	X	X
Were appropriate methods used to statistical combine results for the meta-analyses?	V	V	V	V	V	V	V	V
Was potential impact of RoB in individual studies on the results of the meta-analyses assessed?	V	V	X	V	X	V	V	X
Was RoB in individual studies accounted for when interpreting/discussing the results?	V	V	X	V	X	V	V	X
Was a satisfactory explanation for, and discussion of, any heterogeneity observed in the results provided?	V	V	V	V	X	V	X	V
Was an adequate investigation of publication bias carried out?	V	V	V	X	V	X	V	X
Did the authors report any potential sources of conflict of interest?	V	V	V	V	V	V	X	V
**AMSTAR 2 score**	**Moderate**	**Moderate**	**Low**	**Moderate**	**Low**	**Moderate**	**Moderate**	**Low**

### Quality of meta-analyses

According to AMSTAR 2 scoring, five meta-analyses were of moderate quality, and three meta-analyses were of low quality (Table 2). The meta-analyses by Conn [18] was deemed of low quality due to study selection not being performed in duplicate, inadequate description of the included studies, and inadequate assessment of risk of bias. The author investigated the effect of random versus not random allocation sequence, but did not report assessing the effects of unconcealed allocation, lack of blinding of patients and assessors, or selective reporting. The potential impact of risk of bias on the results needs to be considered in order to assess the strength of the reported evidence. Similarly, Rethorst et al. [17] reported the effect of random allocation sequence and unconcealed allocation, but did not investigate the other factors that might cause risk of bias. In addition, this meta-analysis was assessed as low quality due to study selection and data extraction not being performed in duplicate, and not investigating publication bias. The meta-analyses by Gordon et al. [26] was rated as low quality, because they did not report on which specific population they targeted for their research. In addition, the study selection was not performed in duplicate, the potential impact of risk of bias was not assessed or discussed, and the significant heterogeneity between the studies was not discussed or explained.

### Effect of exercise interventions on depressive symptoms

#### Overall effect

**Fig. 2.**
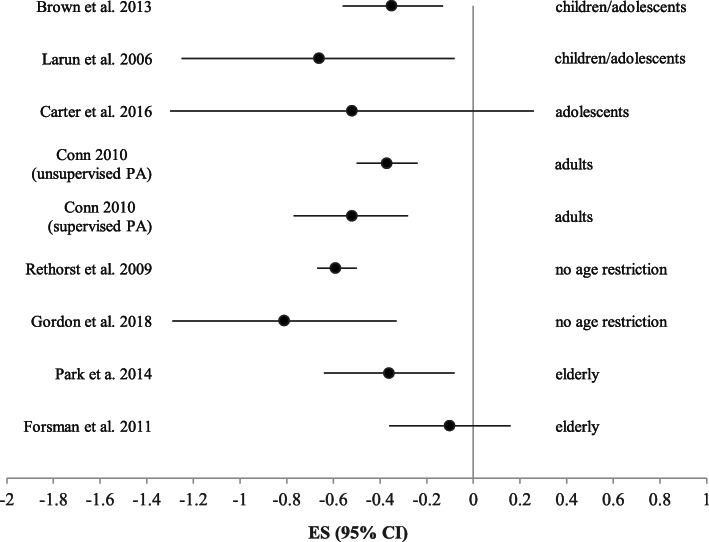
Forest plot of effect sizes (95% confidence interval) of exercise interventions on depression found by the included meta-analyses

Table 3 and Figure 2 show the results of the included meta-analyses. These studies used a similar method to calculate aggregate effects: standardized mean differences (SMDs) using a random effects model. Five meta-analyses [17–19, 27, 28] weighted the studies by the inverse of variance to give larger samples more influence. For Carter et al. [24], Forsman et al. [20], and Gordon et al. [26] it was unclear how the included studies were pooled.

**Table 3:** Results of meta-analyses included in the current study

Review	Comparison	***N*** RCTs	% unique studies	***N*** participants	Effect on depressive symptoms	95% CI	***I***^***2***^
Brown et al. 2013 [19]	Exercise vs control	5	80	581	Hedges’ g = − 0.35**	-0.56, − 0.13	60%
Carter et al. 2016 [25]	Exercise vs control	5	100	1157	SMD = − 0.52	-1.30, 0.26	83%
Conn 2010 [18]	Supervised exercise vs control	38	83	1598	SMD = − 0.37***	− 0.50, − 0.24	45%
	Unsupervised exercise vs control	22		1081	SMD = − 0.52***	− 0.77, − 0.28	66%
Forsman et al. 2011 [20]	Exercise vs control	3	67	277	SMD = − 0.10	− 0.36, 0.16	0%
Gordon et al. 2018 [26]	Exercise vs control	15	93	550	Hedges’ d = −0.81***	−1.29, − 0.33	NP
Larun et al. 2006 [27]	Vigorous exercise vs control	5	40	145	SMD = −0.66*	−1.25, − 0.08	80%
Park et al. 2014 [28]	Exercise vs control	18	94	3297	SMD = −0.36*	−0.64, − 0.08	93%
Rethorst et al. 2009 [17]	Exercise vs control	40	65	2408	Hedges’ g = −0.59*	−0.67, − 0.5	NP

*RCTs* randomized controlled trials, *Exercise* physical activity, *SMD* standardized mean differences, *Q* denotes instances in which Q-value and significance was provided in absence of *I*^*2*^ statistic, *NP* not provided

*p < .05. **p < .01. ***p < .001

Two meta-analyses found non-significant effects of the exercise intervention on subsequent depressive symptoms [25, 27]. These were the meta-analyses by Carter et al. [25] and Forsman et al. [20]. The study by Carter et al. had a comparable effect size (− 0.52) to the other included meta-analyses, but a much larger confidence interval. This may be due to the larger heterogeneity in exercise intervention in this study compared to the other meta-analyses, as interventions varied from yoga to dance and other sports. The meta-analysis by Forsman et al. contained a low number of RCTs (*n* = 3) and total number of participants (*n* = 277), and specifically targeted the elderly population. This relatively lower power and selective sample may have contributed to the divergent effect size in this study. In contrast, six meta-analyses found a significant effect of exercise interventions in reducing depressive symptoms, with effect sizes ranging from − 0.35 to − 0.81. These meta-analyses varied substantially in size and the targeted age-group. The two meta-analyses containing the most studies across the widest age-range were those by Conn [18] and Rethorst et al. [17]. Notably, both meta-analyses were assessed to be of low quality. In addition, Conn reported on high publication bias and high heterogeneity among the studies. Moderation analyses showed that this heterogeneity might be caused by publication status, presence of funding, treatment allocation, and intervention characteristics. Unpublished and unfunded studies reported larger effect sizes than published and funded studies, and exercise interventions without random assignment of participants to treatment and control conditions reported larger effect sizes than those where assignment was truly random. As the primary studies were found to be heterogeneous and their risk of bias was not fully assessed, caution is warranted when interpreting the findings of this meta-analysis.

Rethorst and colleagues reported on high heterogeneity and investigated potential modifiers for all included studies (both clinical and general populations), but did not report on these separately for the general populations. For the overall population, intervention characteristics were found to be significant moderators. In addition, the effects sizes of studies that used adequate concealment and adequate intent to treat were larger than those studies that did not. As risk of bias and publication bias were not fully assessed in this meta-analysis, these results should likewise be interpreted with caution.

The other rather large study including a wide age-range, was by Gordon et al. [26]. This study found the largest effect size of 0.81. A possible explanation for why Gordon et al. found a substantially larger effect of exercise on depression compared to the other studies, is that this study focused on resistance training as an exercise intervention and excluded all other types of exercise. Despite of the focus on resistance training, this meta-analysis found high heterogeneity for all included studies (both clinical and healthy population), but did not report on these separately for the healthy populations. Moderation analyses showed that effects were significantly smaller when outcome allocation and/or assessment was blinded compared with when outcome allocation and/or assessment was not blinded. Notably, this study was also deemed of low quality as the impact of risk of bias and heterogeneity on the overall results were not adequately discussed or explained. Therefore, the true cause for this relatively large effect size remains unclear and, also for this study, we should be thoughtful when interpreting the results.

#### Effects in different age-groups

The three meta-analyses focusing on both children and adolescents included a total of 14 studies. The effect sizes for this age-group were similar to the studies conducted by Conn [18], Gordon et al. [26], and Rethorst et al. [17] on the wider age-ranges (mean age of 9–81 years).

Of the meta-analyses focusing on children and adolescents, Larun et al. [27] found the largest effect size (− 0.66; 95% CI: − 1.25, − 0.08) of vigorous exercise interventions compared to no intervention on depressive symptoms in 145 children and adolescents. However, all primary studies in this meta-analysis were of low quality and high heterogeneity was found. This heterogeneity was likely caused by type of intervention, as resistance training was found to have a larger effect than aerobic training. It is important to note that the number of included studies in these categories was too small to draw any affirmative conclusions on this. The lack of testing for publication bias additionally hampers the findings in this meta-analysis.

In contrast to the high effect size found by Larun et al. [27], Brown et al. [19] found a rather small effect size (− 0.35; 95% CI = − 0.56, − 0.13) when investigating the effect of five exercise intervention studies on depressive symptoms in a total of 581 children and adolescents. The higher effect size found by Larun and colleagues might be explained by the lower quality and high heterogeneity of the included studies. Indeed, Brown and colleagues rated their included primary studies to be of higher quality (high = 3, moderate = 1, low = 1). However, heterogeneity among studies was still found to be significant in this meta-analysis. Both Larun et al. and Brown et al. included relatively few RCTs and a lower total number of participants. Combined with the large heterogeneity, this may have contributed to an over- and underestimation of the effect. Moderator analyses were performed for all included studies (both RCTs and other designs), but did not report the results separately for the RCTs.

Carter et al. [25] examined 5 intervention studies including 1157 adolescents and found a moderate nonsignificant effect for exercise on depressive symptoms in the general population (effect size = − 0.52; 95% CI = − 1.30, 0.26). Interestingly, the authors also investigated the effect of exercise interventions on depressive symptoms in clinical samples in the same article and found a moderate significant effect for this group. They argued that the studies on the general population were of lower methodological quality and that heterogeneity among the studies was high. For the overall population (both clinical and general samples), differences in study quality might have contributed to the high heterogeneity. There appeared to be no publication bias after inspection of the funnel plot.

Two meta-analyses investigated older adults and included a total of 20 studies. The meta-analysis by Forsman et al. [20] examined the effect of several psychosocial interventions on depressive symptoms in older people and included only three interventions regarding exercise. The authors found no significant effect of exercise on depressive symptoms (effect size = − 0.10, 95% CI = -0.36, 0.16). There was no heterogeneity among the studies. The authors, however, did not report on the quality of the separate studies nor did they test for publication bias.

The meta-analysis conducted by Park et al. [28] included many more studies (*n* = 18) and found a significant effect of exercise on depressive symptoms in the elderly (effect size = − 0.36; 95% CI = -0.64, − 0.08). The authors concluded that the quality of almost all included studies was high. They reported a significant publication bias and high heterogeneity across the studies. However, possible causes of this heterogeneity were not investigated. When taking a closer look at the included studies, it was found that three studies comprised persons with (minor) depression at baseline. Nevertheless, we decided to include this meta-analysis in this systematic review as the vast majority of the studies were conducted in general/at-risk populations, and these findings were similar to those in the clinical samples.

#### Role of sex and age

Conn [18] and Gordon et al. [26] attempted to statistically test the moderating effect of sex and age on the effectiveness of exercise interventions on depressive symptoms, and found no such effect. For age, this is in line with the above observation that exercise interventions reduced depressive symptoms across the entire age-range. However, moderator analyses in meta-analyses have methodological issues and are limited in power. Therefore, no firm conclusions can be drawn from these findings.

#### Role of exercise characteristics

Larun et al. [19] explored the effect of exercise intensity on depressive symptoms within two RCTs (of moderate and low quality) that compared vigorous exercise to low intensity exercise. The authors concluded that exercise intensity made no difference in depression scores.

The finding that low intensity exercise is at least as effective as high intensity exercise for depressive symptoms was corroborated by the results of Conn [18], who found that low intensity interventions resulted in a larger decrease in depressive symptoms than moderate intensity interventions. In addition, Conn also found that interventions were most effective when they were not only focused on endurance training. The addition of either flexibility or resistance training was associated with a further decrease in depressive symptoms. For unsupervised exercise, the results showed that a shorter duration of the intervention improved depressive symptoms and that interventions recommending more minutes per week had a smaller effect on decreasing depression.

The findings by Conn were largely corroborated by the meta-analysis by Gordon et al. [26] and Rethorst et al. [17]. Gordon et al. found that shorter resistance exercise training sessions (< 45 min) resulted in larger reductions in depressive symptoms than longer session durations. Rethorst et al. suggested that shorter interventions and bout durations resulted in greater effects than longer interventions and bout durations, and a regimen of combined aerobic and resistance training had larger effects than one mode of exercise. However, as Conn’s, Gordon’s, and Rethorst’s conclusions were based on moderation analyses across studies, these results are at most indirect evidence.

## Discussion

This systematic review included eight meta-analyses that comprised a total of 134 individual studies with little overlap. The results indicate that exercise interventions may have a moderate effect (effect sizes ranging from − 0.10 to − 0.81) on decreasing depressive symptoms in the general population. Exercise seems to have a beneficial effect on depressive symptoms in children, adolescents, adults, and the elderly based on meta-analyses focusing on different age groups. No evidence was available for onset of depression. In addition, there was limited evidence regarding effects of sex, and some low-quality evidence suggests that low intensity exercise may be as effective as high intensity exercise in lowering depression scores.

Although the meta-analyses varied substantially in size and the targeted age-group, most of the included articles indicated that exercise interventions may cause a decrease in future depressive symptoms [17–19, 27, 28], in line with the results of the meta-analysis of meta-analyses by Rebar et al. [21], the systematic review of prospective studies by Mammen and Faulkner [13], and the meta-analysis of prospective studies by Schuch et al. [14]. However, there were two studies that did not find these effects to be significant [25, 27]. The authors of these studies attributed these null-findings to the low quality, high heterogeneity, and/or the small number of included studies. The meta-analysis by Carter et al. [25] had a moderate effect size that was comparable to the other included studies. However, the confidence interval was much larger compared to the other studies, possibly due to the larger heterogeneity in exercise intervention in this study compared to the other meta-analyses, as well as to possible other sources of heterogeneity and the low quality of the included individual RCTs. The meta-analysis by Forsman et al. [20] reported the lowest effect size, likely due to the small number of included studies and total number of participants, the low quality of the studies, and the high heterogeneity. When interpreting the results, it should also be taken into account that three meta-analyses containing studies across the widest age range [17, 18, 26] were of low quality according to AMSTAR 2 scoring, mainly due to inadequate assessment of risk of bias, adding uncertainty to the estimated effects of exercise on depressive symptoms. In addition, heterogeneity of primary studies was often found to be high, likely caused by differences in risk of bias between the studies and differences in exercise characteristics. As heterogeneity among studies was high and risk of bias was often not adequately assessed, the overall conclusion that exercise may be effective in decreasing depressive symptoms is stated with caution.

The systematic review conducted by Mammen and Faulkner [13] indicated that some studies found the prospective relationship between exercise and onset of depression to be specific to women and girls, and other studies found that this relationship might not apply to older adults. Unfortunately, the included meta-analyses reported scarcely on the role of sex and age. However, when comparing the meta-analyses on children/adolescents and the elderly [19, 25, 27] to the meta-analyses on the broader age-ranges [17, 18], the effect sizes seemed to be similar. Interestingly, two of the studies targeting children/adolescents reported one of smallest effect sizes (Brown et al. [19]) and one the largest effect size (Larun et al. [27]). Both studies included relatively few RCTs, a low total number of participants, and a large heterogeneity among studies, which might have contributed to an over- and underestimation of the effect of exercise on depressive symptoms. Nonetheless, taken together, also for children/and adolescents the effect of exercise on depressive symptoms appears to be moderate. Comparable results were found by a recent umbrella systematic review conducted by Dale et al. [29], concluding that exercise was associated with a decrease in depression in children and youth. More RCTs and meta-analyses ought to examine sex and age effects in this field, but the scarce evidence in this systematic review suggests that exercise may be effective in decreasing depressive symptoms across a wide age-range.

Regarding exercise characteristics, only the meta-analysis by Larun [27] investigated the effect of exercise intensity on depressive symptoms within RCTs. The authors concluded from two trials that compared vigorous exercise to low intensity exercise that exercise intensity made no difference in depression scores. The meta-analysis by Conn [18] investigated the moderating effect of exercise characteristics across RCTs and these findings also suggested that even low levels of exercise were effective in decreasing depressive symptoms. However, Larun based these conclusions on a limited amount of studies and Conn used indirect methods for analyses. Interestingly, compared to the other included meta-analyses, the study by Gordon et al. [26] that focused solely on resistance exercise training found the largest effect size of 0.81. Whether resistance training is more effective than other types of exercise, and whether other characteristics play a role in the effectivity of exercise on depression, remains to be elucidated. Dale and colleagues [29] investigated similar potential moderators of age, sex, and exercise characteristics, and reached the same conclusion that there is not enough consistent or conclusive evidence that these factors moderate the effect of exercise on depressive symptoms in children and youth.

This systematic review was limited by the varying quality of the included meta-analyses, as risk of bias and publication bias of the studies were not always assessed. In addition, the meta-analyses were limited by the included primary studies, which were highly heterogeneous, especially concerning study quality and exercise characteristics. For instance, an important source of heterogeneity is differences in control comparison. Conn [18], Larun et al. [27], and Park et al. [28] allowed non-exercise interventions, such as psychosocial interventions as a control condition, which may not be comparable to wait-list or no-treatment control conditions. In fact, it has been found that waiting-list control conditions increases the contrast in RCTs as compared to active attention control conditions [30]. However, the effect sizes found by the meta-analyses including studies with active attention control conditions do not appear to be smaller than the effect sizes found by the other meta-analyses. Therefore, these differences in control conditions likely do not affect the results of this study significantly. In addition, although meta-analyses that specifically focused on populations with acute or chronic physical or mental illnesses were excluded, there were meta-analyses that included some individual studies with chronically ill patients. These sources of heterogeneity may have contributed to differences in the found effect sizes. Another concern is that the included meta-analyses were unable to answer our research question directly [31]. The aim of this study was to investigate the effect of exercise interventions on depression from a preventive perspective. However, many of the primary studies included in the meta-analyses focused on populations that were at risk and/or might not have been entirely free of depression. In addition, although studies were excluded that focused on clinically depressed patients, the distinction between clinical and subclinical symptoms was based on an arguably arbitrary cut-off score measured by different instruments, adding another source of heterogeneity to the findings. Furthermore, all of the studies employed depressive symptoms rather than onset of depression as outcome. Therefore, we conclude there is a lack of evidence for true prevention of depression by exercise. However, the meta-analyses included in this systematic review indicate that early depressive symptoms may be decreased by exercise interventions.

## Conclusion

This systematic review was based on eight meta-analyses showing little overlap in 134 included individual RCTs, and provides the most comprehensive overview up to date on the effectiveness of exercise interventions in decreasing depressive symptoms in the general population. Further high quality research is needed, especially concerning onset of depression outcome, and the effects of sex, age, and characteristics of exercise. Nonetheless, the evidence from this study suggests that exercise has a moderate effect on decreasing symptoms of depression in the general population across a wide age-range. As exercise has shown to be beneficial for many health aspects and is an inexpensive, easily modifiable lifestyle factor with virtually no negative side effects, the evidence from this systematic review might help to develop early intervention strategies for depression.

### Supplementary information

**Additional file 1: Supplementary Table 1.** List of studies excluded from systematic review.

## Data Availability

Not applicable.

## Appendix

### Search strings

1. PubMed
  Depression [MH] OR Depress* OR Mood disorder [MH] OR “Mood disorder” OR “Depressive disorder” OR Dysthymi* OR “Low Mood” AND Exercise [MH] OR exercise OR aerobic* OR running [MH] OR running OR walking [MH] OR walk* OR swimming [MH] OR swim* OR sports [MH] OR sport* OR bicycling [MH] OR bicycl* OR cycling OR “physical activity” OR Fitness OR Resistance training [MH] OR “resistance training” OR yoga [MH] OR yoga.
  **Filter:** Limit to meta-analysis.
2. Embase
  Depression OR Depress* OR Mood disorder OR Low Mood OR Dysthymia OR Dysthymi* AND Exercise OR aerobic* OR running OR walking OR walk* OR swimming OR swim* OR sports OR sport* OR bicycling OR bicycl* OR cycling OR physical activity OR Fitness OR resistance training OR yoga.
  **Filter:** Limit to meta-analysis.
3. PsychInfo
  Depression OR Depress* OR Mood disorder OR Low Mood OR Dysthymia OR Dysthymi* AND Exercise OR aerobic* OR running OR walking OR walk* OR swimming OR swim* OR sports OR sport* OR bicycling OR bicycl* OR cycling OR physical activity OR Fitness OR resistance training OR yoga.
  **Filter:** Limit to meta-analysis.
4. The Cochrane database of systematic reviews
  (Depression OR Depress* OR Mood disorder OR Low Mood OR Dysthymia OR Dysthymi*) AND (Exercise OR aerobic* OR running OR walking OR walk* OR swimming OR swim* OR sports OR sport* OR bicycling OR bicycl* OR cycling OR physical activity OR Fitness OR resistance training OR yoga)
  **Filter:** Limit to reviews.

## Abbreviations

AMSTAR 2: A MeaSurement Tool to Assess systematic Reviews, revised instrument 2
RCT: Randomized controlled trial
PICOS: Participants, intervention, comparison, outcome, study design
