# Supplementary material for: Exercise interventions for the prevention of depression: a systematic review of meta-analyses
Source: BMC Public Health. 2020 Aug 18;20:1255. doi: 10.1186/s12889-020-09323-y (PMC7436997; doi:10.1186/s12889-020-09323-y)
Supplement: Supplementary file 1 — Additional file 1: Supplementary Table 1. List of studies excluded from systematic review. [file 12889_2020_9323_MOESM1_ESM.docx]

Additional file 1

| **Supplementary table 1.** List of studies excluded from systematic review. | |
| --- | --- |
| **Reasons for exclusion** | **Study** |
| Inappropriate design e.g. systematic reviews not including meta-analysis or non-intervention focused meta-analyses on prospective data | Cramer et al., 2014 (14)  Korczak et al., 2017 (24)  Mammen and Faulkner, 2013 (29)  Patel et al., 2012 (34)  Rebar et al., 2015 (42) |
| Inappropriate intervention | Gogulla et al., 2012 (20) |
| Non-relevant outcome measures | Gilinsky et al., 2015 (19)  Lindheimer et al., 2015 (25)  Lubans et al., 2012 (28)  Montgomery et al., 2002 (30)  Peñalba et al., 2008 (36)  Puetz et al., 2006 (40)  Raymond et al., 2013 (41)  Windle et al., 2010 (56) |
| Inappropriate sample, e.g. clinical samples | Adamson et al., 2015 (1)  Allison et al., 1995 (2)  Andrews et al., 2012 (3)  Baillot et al., 2018 (4)  Bergenthal et al., 2014 (5)  Blake et al., 2009 (6)  Bridle et al., 2012 (7)  Brown et al., 2017 (8)  Brown et al., 2012 (9)  Carayol et al., 2015 (10)  Catalan-Matamoros et al., 2016 (11)  Cezaretto et al.,2016 (12)  Chi et al., 2013 (13)  Daley et al., 2015 (15)  Dunn et al.,, 2001 (16)  Fabricatore et al., 2011 (17)  Galantino et al., 2008 (18)  Hanson and Jones, 2015 (21)  Heinzel et al., 2015 (22)  Janssen and LeBlanc, 2010 (23)  Liu et al., 2017 (26)  Liu et al., 2015 (27)  North et al., 1990 (31)  North et al., 1989 (32)  Oh et al., 2013 (33)  Pavey et al., 2011 (35)  Pérez-López et al., 2017 (37)  Poquet and Maher., 2015 (38)  Poyatos-León et al., 2017 (39)  Rhyner et al., 2016 (43)  Robertson et al., 2012 (44)  Sjösten and Kivelä, 2006 (45)  Stanton and Reaburn, 2014 (46)  Stathopoulou et al., 2006 (47)  Stich, 1999 (48)  Van Dammen et al., 2018 (49)  Villada et al., 2013 (50)  Wang et al., 2010 (51)  Wang et al., 2013a (52)  Wang et al., 2013b (53)  Wang et al., 2014a (54)  Wang et al., 2014b (55)  Xiang et al., 2017 (57)  Yan et al., 2016 (58)  Yin et al., 2014 (59) |
| Duplicates not detected | Bridle et al., 2012 (7)  Daley et al., 2014 (15)  Patel et al., 2012 (34)  Robertson et al., 2012 (44)  Stathopoulou et al., 2006 (47)  Wang et al., 2010 (51) |

**References of excluded studies**

1. Adamson, B. C., Ensari, I., & Motl, R. W. (2015). Effect of exercise on depressive symptoms in adults with neurologic disorders: a systematic review and meta-analysis. *Archives of Physical Medicine and Rehabilitation*, *96*(7), 1329-1338.

2. Allison, D. B., Faith, M. S., & Franklin, R. D. (1995). Antecedent exercise in the treatment of disruptive behavior: a meta‐analytic review. *Clinical Psychology: Science and Practice*, *2*(3), 279-303.

3. Andrews, N. E., Strong, J., & Meredith, P. J. (2012). Activity pacing, avoidance, endurance, and associations with patient functioning in chronic pain: a systematic review and meta-analysis. *Archives of physical medicine and rehabilitation*, *93*(11), 2109-2121.

4. Baillot, A., Saunders, S., Brunet, J., Romain, A. J., Trottier, A., & Bernard, P. (2018). A systematic review and meta-analysis of the effect of exercise on psychosocial outcomes in adults with obesity: A call for more research. *Mental Health and Physical Activity*, *14*, 1-10.

5. Bergenthal, N., Will, A., Streckmann, F., Wolkewitz, K., Monsef, I., Engert, A., Elter, T., Skoetz, N. (2014). Aerobic physical exercise for adult patients with haematological malignancies. *Cochrane Database of Systematic Reviews,* *1*(11).

6. Blake, H., Mo, P., Malik, S., & Thomas, S. (2009). How effective are physical activity interventions for alleviating depressive symptoms in older people? A systematic review. Clinical rehabilitation, 23(10), 873-887.

7. Bridle, C., Spanjers, K., Patel, S., Atherton, N. M., & Lamb, S. E. (2012). Effect of exercise on depression severity in older people: systematic review and meta-analysis of randomised controlled trials. The British Journal of Psychiatry, 201(3), 180-185.

8. Brown, J., Alwan, N. A., West, J., Brown, S., McKinlay, C. J., Farrar, D., & Crowther, C. A. (2017). Lifestyle interventions for the treatment of women with gestational diabetes. *Cochrane Database of Systematic Reviews*, (5).

9. Brown, J. C., Huedo-Medina, T. B., Pescatello, L. S., Ryan, S. M., Pescatello, S. M., Moker, E., ... & Johnson, B. T. (2012). The efficacy of exercise in reducing depressive symptoms among cancer survivors: a meta-analysis. *PloS one*, *7*(1).

10. Carayol, M., Delpierre, C., Bernard, P., & Ninot, G. (2015). Population‐, intervention‐and methodology‐related characteristics of clinical trials impact exercise efficacy during adjuvant therapy for breast cancer: A meta‐regression analysis. *Psycho‐Oncology*, *24*(7), 737-747.

11. Catalan-Matamoros, D., Gomez-Conesa, A., Stubbs, B., & Vancampfort, D. (2016). Exercise improves depressive symptoms in older adults: An umbrella review of systematic reviews and meta-analyses. Psychiatry research, 244, 202-209.

12. Cezaretto, A., Ferreira, S. R. G., Sharma, S., Sadeghirad, B., & Kolahdooz, F. (2016). Impact of lifestyle interventions on depressive symptoms in individuals at-risk of, or with, type 2 diabetes mellitus: a systematic review and meta-analysis of randomized controlled trials. *Nutrition, Metabolism and Cardiovascular Diseases*, *26*(8), 649-662.

13. Chi, I., Jordan‐Marsh, M., Guo, M., Xie, B., & Bai, Z. (2013). Tai chi and reduction of depressive symptoms for older adults: A meta‐analysis of randomized trials. *Geriatrics & gerontology international*, *13*(1), 3-12.

14. Cramer, H., Lauche, R., & Dobos, G. (2014). Characteristics of randomized controlled trials of yoga: a bibliometric analysis. *BMC complementary and alternative medicine*, *14*(1), 328.

15. Daley, A. J., Foster, L., Long, G., Palmer, C., Robinson, O., Walmsley, H., & Ward, R. (2015). The effectiveness of exercise for the prevention and treatment of antenatal depression: systematic review with meta‐analysis. *BJOG: An International Journal of Obstetrics & Gynaecology*, *122*(1), 57-62.

16. Dunn, A. L., Trivedi, M. H., & O’NEAL, H. A. (2001). Physical activity dose-response effects on outcomes of depression and anxiety. *Medicine & Science in Sports & Exercise*, *33*(6), S587-S597.

17. Fabricatore, A. N., Wadden, T. A., Higginbotham, A. J., Faulconbridge, L. F., Nguyen, A. M., Heymsfield, S. B., & Faith, M. S. (2011). Intentional weight loss and changes in symptoms of depression: a systematic review and meta-analysis. *International journal of obesity*, *35*(11), 1363-1376.

18. Galantino, M. L., Galbavy, R., & Quinn, L. (2008). Therapeutic effects of yoga for children: a systematic review of the literature. *Pediatric Physical Therapy*, *20*(1), 66-80.

19. Gilinsky, A. S., Dale, H., Robinson, C., Hughes, A. R., McInnes, R., & Lavallee, D. (2015). Efficacy of physical activity interventions in post-natal populations: systematic review, meta-analysis and content coding of behaviour change techniques. *Health Psychology Review*, *9*(2), 244-263.

20. Gogulla, S., Lemke, N., & Hauer, K. (2012). Effects of physical activity and physical training on the psychological status of older persons with and without cognitive impairment.

21. Hanson, S., & Jones, A. (2015). Is there evidence that walking groups have health benefits? A systematic review and meta-analysis. *Br J Sports Med*, *49*(11), 710-715.

22. Heinzel, S., Lawrence, J. B., Kallies, G., Rapp, M. A., & Heissel, A. (2015). Using Exercise to Fight Depression in Older Adults. *GeroPsych*.

23. Janssen, I., & LeBlanc, A. G. (2010). Systematic review of the health benefits of physical activity and fitness in school-aged children and youth. *International journal of behavioral nutrition and physical activity*, *7*(1), 40.

24. Korczak, D. J., Madigan, S., & Colasanto, M. (2017). Children’s physical activity and depression: a meta-analysis. *Pediatrics*, *139*(4), e20162266.

25. Lindheimer, J. B., O’Connor, P. J., & Dishman, R. K. (2015). Quantifying the placebo effect in psychological outcomes of exercise training: a meta-analysis of randomized trials. *Sports Medicine*, *45*(5), 693-711.

26. Liu, J., Nie, J., & Wang, Y. (2017). Effects of group counseling programs, cognitive behavioral therapy, and sports intervention on internet addiction in East Asia: a systematic review and meta-analysis. *International journal of environmental research and public health*, *14*(12), 1470.

27. Liu, X., Clark, J., Siskind, D., Williams, G. M., Byrne, G., Yang, J. L., & Doi, S. A. (2015). A systematic review and meta-analysis of the effects of Qigong and Tai Chi for depressive symptoms. *Complementary therapies in medicine*, *23*(4), 516-534.

28. Lubans, D. R., Plotnikoff, R. C., & Lubans, N. J. (2012). A systematic review of the impact of physical activity programmes on social and emotional well‐being in at‐risk youth. *Child and adolescent mental health, 17*(1), 2-13.

29. Mammen, G., & Faulkner, G. (2013). Physical activity and the prevention of depression: a systematic review of prospective studies. *American journal of preventive medicine*, *45*(5), 649-657.

30. Montgomery, P., & Dennis, J. A. (2002). Physical exercise for sleep problems in adults aged 60+. *Cochrane Database of Systematic Reviews*, (4).

31. North, T. C., McCullagh, P & Tran, Z. V. (1990). Effect of exercise on depression. *Exercise and sport sciences reviews*, *18*(1), 379-416.

32. North, T. C. (1989). The effect of exercise on depression: A meta-analysis. (1989). *Dissertation Abstracts International, 49*(11-B), 5027-5028.

33. Oh, B., Choi, S. M., Inamori, A., Rosenthal, D., & Yeung, A. (2013). Effects of qigong on depression: a systemic review. *Evidence-Based Complementary and Alternative Medicine*, *2013*.

34. Patel, N. K., Newstead, A. H., & Ferrer, R. L. (2012). The effects of yoga on physical functioning and health related quality of life in older adults: a systematic review and meta-analysis. *The journal of alternative and complementary medicine*, *18*(10), 902-917.

35. Pavey, T. G., Taylor, A. H., Fox, K. R., Hillsdon, M., Anokye, N., Campbell, J. L., Foster, C., Green, C., Moxham, T., Mutrie, N., Searle, J., Trueman, P., & Taylor, R. S. Effect of exercise referral schemes in primary care on physical activity and improving health outcomes: Systematic review and meta-analysis. (2011). *BMJ: British Medical Journal, 343*(7831), 1-14.

36. Peñalba, V., McGuire, H., & Leite, J. R. (2008). Psychosocial interventions for prevention of psychological disorders in law enforcement officers. *Cochrane Database of Systematic Reviews*, (3).

37. Perez-Lopez, F. R., Martinez-Dominguez, S. J., Lajusticia, H., Chedraui, P., & Project, T. H. O. S. A. (2017). Effects of programmed exercise on depressive symptoms in midlife and older women: a meta-analysis of randomized controlled trials. *Maturitas*, *106*, 38-47.

38. Poquet, N., & Maher, C. G. (2015). Exercise for the management of depression (PEDro synthesis). *Br J Sports Med*, *49*(24), 1595-1595.

39. Poyatos‐León, R., García‐Hermoso, A., Sanabria‐Martínez, G., Álvarez‐Bueno, C., Cavero‐Redondo, I., & Martínez‐Vizcaíno, V. (2017). Effects of exercise‐based interventions on postpartum depression: A meta‐analysis of randomized controlled trials. *Birth*, *44*(3), 200-208.

40. Puetz, T. W., O'Connor, P. J., & Dishman, R. K. (2006). Effects of chronic exercise on feelings of energy and fatigue: a quantitative synthesis. *Psychological bulletin*, *132*(6), 866.

41. Raymond, M. J., Bramley-Tzerefos, R. E., Jeffs, K. J., Winter, A., & Holland, A. E. (2013). Systematic review of high-intensity progressive resistance strength training of the lower limb compared with other intensities of strength training in older adults. *Archives of physical medicine and rehabilitation*, *94*(8), 1458-1472.

42. Rebar, A. L., Stanton, R., Geard, D., Short, C., Duncan, M. J., & Vandelanotte, C. (2015). A meta-meta-analysis of the effect of physical activity on depression and anxiety in non-clinical adult populations. *Health psychology review*, *9*(3), 366-378.

43. Rhyner, K. T., & Watts, A. (2016). Exercise and depressive symptoms in older adults: a systematic meta-analytic review. *Journal of aging and physical activity*, *24*(2), 234-246.

44. Robertson, R., Robertson, A., Jepson, R., & Maxwell, M. (2012). Walking for depression or depressive symptoms: a systematic review and meta-analysis. *Mental health and physical activity*, *5*(1), 66-75.

45. Sjösten, N., & Kivelä, S. L. (2006). The effects of physical exercise on depressive symptoms among the aged: a systematic review. *International Journal of Geriatric Psychiatry: A journal of the psychiatry of late life and allied sciences*, *21*(5), 410-418.

46. Stanton, R., & Reaburn, P. (2014). Exercise and the treatment of depression: a review of the exercise program variables. *Journal of Science and Medicine in Sport*, *17*(2), 177-182.

47. Stathopoulou, G., Powers, M. B., Berry, A. C., Smits, J. A., & Otto, M. W. (2006). Exercise interventions for mental health: a quantitative and qualitative review. *Clinical psychology: Science and practice*, *13*(2), 179-193.

48. Stich, F. A. (1999). A meta-analysis of physical exercise as a treatment for symptoms of anxiety and depression. (1999). *Dissertation Abstracts International: Section B: The Sciences and Engineering, 59*(8-B), 4487-4487.

59. Van Dammen, L., Wekker, V., de Rooij, S. R., Groen, H., Hoek, A., & Roseboom, T. J. (2018). A systematic review and meta‐analysis of lifestyle interventions in women of reproductive age with overweight or obesity: the effects on symptoms of depression and anxiety. *Obesity Reviews*, *19*(12), 1679-1687.

50. Villada, F. A., Velez, E. F., & Baena, L.Z. Physical exercise and depression in the elderly: a systematic review (Provisional abstract). (2013). Revista Colombiana de Psiquiatria, 42(2), 198-211.

51. Wang, C., Bannuru, R., Ramel, J., Kupelnick, B., Scott, T., & Schmid, C. H. (2010). Tai Chi on psychological well-being: systematic review and meta-analysis. *BMC complementary and alternative medicine*, *10*(1), 23.

52. Wang, C. W., Chan, C. L. W., Ho, R. T., Tsang, H. W., Chan, C. H. Y., & Ng, S. M. (2013). The effect of qigong on depressive and anxiety symptoms: a systematic review and meta-analysis of randomized controlled trials. *Evidence-Based Complementary and Alternative Medicine*, *2013*.

53. Wang, F., Man, J. K., Lee, E. K. O., Wu, T., Benson, H., Fricchione, G. L., ... & Yeung, A. (2013). The effects of qigong on anxiety, depression, and psychological well-being: a systematic review and meta-analysis. *Evidence-Based Complementary and Alternative Medicine*, *2013*.

54. Wang, F., Lee, E. K. O., Wu, T., Benson, H., Fricchione, G., Wang, W., & Yeung, A. S. (2014). The effects of tai chi on depression, anxiety, and psychological well-being: a systematic review and meta-analysis. *International journal of behavioral medicine*, *21*(4), 605-617.

55. Wang, Y. Y., Chang, H. Y., & Lin, C. Y. (2014). Systematic review of yoga for depression and quality of sleep in the elderly. *Hu Li Za Zhi*, *61*(1), 85.

56. Windle, G., Hughes, D., Linck, P., Russell, I., & Woods, B. (2010). Is exercise effective in promoting mental well-being in older age? A systematic review. *Aging & mental health*, *14*(6), 652-669.

57. Xiang, Y., Lu, L., Chen, X., & Wen, Z. (2017). Does Tai Chi relieve fatigue? A systematic review and meta-analysis of randomized controlled trials. *PloS one*, *12*(4).

58. Yan, S., Jin, Y., Oh, Y., & Choi, Y. (2016). Effect of exercise on depression in university students: a meta-analysis of randomized controlled trials.

59. Yin, J., & Dishman, R. K. (2014). The effect of Tai Chi and Qigong practice on depression and anxiety symptoms: a systematic review and meta-regression analysis of randomized controlled trials. *Mental Health and Physical Activity*, *7*(3), 135-146.
